# Sino-US-DrugQA: A benchmark for evaluating large language models in cross-jurisdictional pharmaceutical regulation

**DOI:** 10.1371/journal.pone.0343858

**Published:** 2026-09-15

**Authors:** Xuejing Fu, Zhen Chen, Wentao Lu

**Affiliations:** 1 Information Application Research Center of Shanghai Municipal Administration for Market Regulation, Shanghai, China; 2 Shanghai Artificial Intelligence Research Institute Co., Ltd., Shanghai, China; Philadelphia University, JORDAN

## Abstract

Cross-jurisdictional pharmaceutical compliance requires comparison of regulatory requirements across administrative systems such as the US Food and Drug Administration and China's National Medical Products Administration. Although large language models (LLMs) are increasingly explored for healthcare and regulatory applications, their performance in cross-jurisdictional pharmaceutical regulation has not been systematically evaluated using a dedicated benchmark. We introduce Sino-US-DrugQA, a bilingual multiple-choice benchmark covering Monolingual, Comparative, and Parallel regulatory question–answer tasks. The 11,871 candidate items underwent deterministic structural validation and full-dataset semantic quality screening, followed by risk-stratified independent review of 1,432 items by two regulatory experts. The final release comprised 11,444 items, including 10,122 classified as pass and 1,322 as borderline. Among 500 items sampled from the semantic screen-negative population, 18 were subsequently classified as material errors, corresponding to an observed residual material-error proportion of 3.60% (Wilson 95% confidence interval, 2.29%–5.62%). Four representative LLMs—gpt-5.6-terra, gemini-3.6-flash, deepseek-v4-flash, and qwen-3.5-max—were evaluated under a standardized zero-shot protocol. Overall accuracy ranged from 83.21% to 86.43%. Comparative accuracy was consistently lower than Monolingual accuracy, with absolute differences of 4.42–8.98 percentage points across models. The two highest-scoring models, GPT and Gemini, did not differ significantly after adjustment for multiple comparisons. These results indicate that explicit comparison across non-equivalent regulatory systems remains more challenging than single-jurisdiction question answering. Sino-US-DrugQA provides a validated and reproducible resource for evaluating bilingual regulatory reasoning. The findings support further investigation of expert-supervised decision-support workflows rather than autonomous regulatory interpretation. The stable dataset release and evaluation resources are available at https://github.com/DodgeLU/Sino-US-DrugQA.

## 1. Introduction

The globalization of pharmaceutical research and development has necessitated harmonization of regulatory standards across major jurisdictions [1,2]. Despite efforts by organizations such as the International Council for Harmonisation of Technical Requirements for Pharmaceuticals for Human Use (ICH), significant disparities persist between the regulatory frameworks of the United States Food and Drug Administration (FDA) and China's National Medical Products Administration (NMPA) [3,4]. For multinational pharmaceutical enterprises, submissions in both jurisdictions require provision-level comparison of Title 21 of the Code of Federal Regulations (CFR) and Chinese pharmaceutical regulations. Such analysis extends beyond literal translation to regulatory intent, procedural timelines, and jurisdiction-specific technical requirements.

Currently, cross-jurisdictional compliance relies heavily on manual gap analyses performed by regulatory affairs (RA) professionals [5,6]. This work is resource-intensive and difficult to maintain consistently as regulatory frameworks evolve, particularly in China [7]. The emergence of artificial intelligence (AI) technologies, particularly large language models (LLMs), has shown promise in supporting clinical decision-making and regulatory tasks [8,9]. LLMs can process multilingual text and generate structured responses, suggesting potential applications in regulatory intelligence, drafting assistance, and preliminary compliance screening [10]. However, whether such capabilities extend reliably to cross-jurisdictional regulatory comparison remains an open question.

Deploying LLMs in cross-jurisdictional regulatory compliance presents unique challenges that distinguish this setting from general medical or legal question answering. Unlike clinical diagnosis or single-jurisdiction legal interpretation, cross-jurisdictional regulatory reasoning requires models to operate across partially overlapping but non-isomorphic administrative systems. Models must interpret multilingual regulatory text, align related but non-equivalent concepts across systems—for example, an Investigational New Drug (IND) application in the United States and clinical trial approval in China—and compare how obligations differ in scope, procedure, and technical specificity. If accepted without verification, incorrect comparisons could misinform preliminary regulatory analysis and subsequent decision-making. Consequently, the reliability of LLMs in this specific cross-jurisdictional regulatory setting remains a critical, yet insufficiently validated, variable.

A major barrier to evaluating this reliability is the lack of specialized benchmarks that reflect cross-jurisdictional pharmaceutical regulation. Existing legal natural language processing (NLP) benchmarks, such as LawBench [11] and LexGLUE [12], primarily evaluate monolingual statutory interpretation, case law reasoning, or judgment prediction within a single legal system. Similarly, medical benchmarks such as MedMCQA [13] emphasize clinical knowledge rather than administrative regulation. Multilingual legal benchmarks have begun to examine reasoning across languages [14], but these resources do not specifically assess comparison of non-equivalent pharmaceutical regulatory requirements between the United States and China.

To address this gap, we introduce Sino-US-DrugQA, a bilingual benchmark for evaluating LLMs in cross-jurisdictional pharmaceutical regulation. Rather than proposing a new model or optimization strategy, this study contributes a validated dataset and a standardized evaluation framework. Before release, the candidate dataset underwent deterministic structural validation, full-dataset semantic quality screening, and risk-stratified expert review to identify and exclude structurally invalid or materially flawed items. Four representative LLMs were then evaluated under a common zero-shot protocol across task types, languages, regulatory object domains, and functional subdomains. This design provides a reproducible baseline for regulatory AI research [15] while supporting cautious interpretation of model outputs under expert supervision. To illustrate the types of provision-level regulatory divergence represented in the benchmark, Table 1 presents selected items comparing US FDA and China NMPA requirements across key compliance domains.

**Table 1:** Selected benchmark items illustrating provision-level regulatory divergence between the United States and China.

Regulatory domain	Regulatory requirement	US FDA	China NMPA	Source question ID
Clinical Trial Applications	Review timeline for clinical trial applications	No fixed timeline specified; review process includes treatment protocols and risk-benefit analyses	60 working days	CO_P_00014_003
Personnel Qualifications	Educational and experience requirements for quality roles	General principles: appropriate education, training, and experience combination	Specific requirements: bachelor's degree in specific fields, 5 years experience (3 years in specific areas)	CO_P_00001_004
Production Facilities	Facility requirements for blood product manufacturing	Emphasis on facility design to prevent contamination through proper flow and separation	Independent buildings with dedicated equipment required	CO_P_00001_005
Raw Material Inspection	Inspection procedures for rejected materials	Quarantine systems for rejected in-process materials	Detailed plasma source verification, mandatory retesting, and destruction of non-compliant materials	CO_P_00001_002
Packaging Requirements	Tamper-evident packaging for over-the-counter (OTC) drugs	Explicit requirement for “distinctive by design” packaging features	Focus on plasma handling and cross-contamination prevention; no specific distinctive design requirements	CO_P_00001_001

## 2. Materials and methods

### 2.1 Regulatory data sources

This retrospective benchmarking study was constructed using authoritative regulatory documents issued by the NMPA and the US FDA, with document versions valid as of December 2025. To ensure comprehensive coverage of regulatory requirements, we selected documents spanning the full pharmaceutical product lifecycle, from clinical trial authorization to post-market surveillance.

The corpus comprised 134 key regulations from the NMPA and 195 documents from Title 21 of the US Code of Federal Regulations (CFR). Table 2 summarizes the primary regulatory sources used in dataset construction, illustrating functional correspondence across regulatory domains rather than asserting one-to-one legal equivalence between the two jurisdictions.

**Table 2:** Key regulatory documents and sources included in the Sino-US-DrugQA dataset.

Regulatory Domain	US Source (FDA)	China Source (NMPA)
General Administration	21 CFR Part 1 (General Enforcement Regulations)	Drug Administration Law of the People's Republic of China (2019 Revision)
Clinical Trials (GCP)	21 CFR Part 312 (IND), Part 50 (Protection of Human Subjects)	Good Clinical Practice (GCP) (2020), Measures for Administration of Drug Registration
Manufacturing (GMP)	21 CFR Part 210, 211 (current Good Manufacturing Practice (cGMP) for Finished Pharmaceuticals)	Good Manufacturing Practice (GMP) (2010 Revision) and Appendices
Non-Clinical (GLP)	21 CFR Part 58 (Good Laboratory Practice)	Good Laboratory Practice (GLP) (2017)
Labeling & Packaging	21 CFR Part 201 (Labeling)	Provisions on the Administration of Pharmaceutical Directions and Labels
Post-Market Surveillance	21 CFR Part 314 (Postmarketing Reporting)	Measures for Administration of Adverse Drug Reaction Monitoring and Reporting

The cited regulatory materials served as the source basis for the reference answers in the dataset. Subsequent construction and validation steps retained item-level metadata linking each question–answer pair to its corresponding source provisions.

### 2.2 Dataset construction pipeline

#### 2.2.1. Candidate item construction.

**Fig 1:**
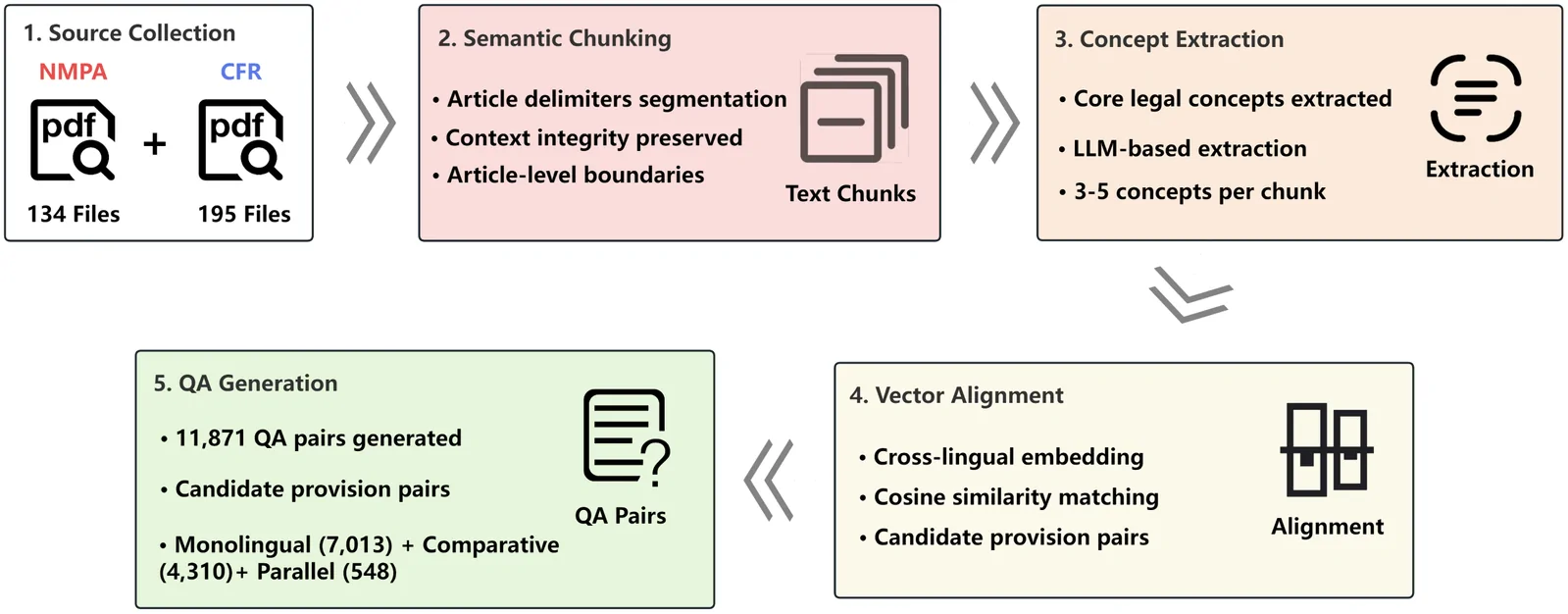
Workflow of the Sino-US-DrugQA dataset construction process, from source collection to candidate question-answer pair generation.

The Sino-US-DrugQA candidate dataset was constructed through a five-stage large language model (LLM)-assisted workflow (Fig 1) designed to balance scalability with regulatory traceability. DeepSeek-V3.2 in chat mode was used during concept extraction and candidate-item drafting. Each candidate item retained links to its source provisions and subsequently underwent the structured quality-validation process described in Section 2.3.

1. **Source Collection:** Authoritative regulatory texts were collected from official NMPA and electronic Code of Federal Regulations (eCFR) portals, ensuring traceability to publicly accessible, regulator-issued source documents. All regulatory documents used in this study were obtained from publicly accessible official sources, and their collection and use complied with the respective terms and conditions governing public regulatory information. No restricted, proprietary, or personally identifiable data were used in this study.
2. **Semantic Chunking:** Documents were segmented into provision-level semantic chunks using explicit article delimiters (e.g., “Article 12,” “§ 211.68”) to preserve contextual integrity and avoid cross-article information leakage.
3. **Concept Extraction:** Core regulatory concepts were operationalized at the chunk level as concise, compliance-relevant semantic units. Specifically, for each semantic chunk, DeepSeek-V3.2 (chat mode, temperature = 0.1, top-p = 1.0) was used to identify a small set of key regulatory concepts (typically 3–5 short English phrases) representing entities, obligations, standards, or procedural requirements expressed in the provision. These concepts are intended to function as intermediate semantic representations that facilitate cross-jurisdictional comparison, rather than as formal legal interpretations. Only chunks with successfully extracted non-empty concept sets were retained for downstream alignment.
4. **Vector Alignment:** To enable cross-jurisdictional comparative reasoning, extracted concept sets were converted into embedding representations and matched across jurisdictions within the same regulatory domain. Concept lists were formatted as comma-separated strings (e.g., “concept1, concept2, concept3”) and prefixed with “search_document:” before being encoded using nomic-embed-text:v1.5. Cosine similarity was used to identify candidate pairs of functionally corresponding provisions. For each source chunk, the highest-scoring cross-jurisdictional match was selected, and only pairs meeting the predefined minimum cosine-similarity threshold (≥ 0.60) were retained for downstream use. This alignment step served solely as a retrieval and pairing mechanism and did not determine regulatory equivalence or answer correctness.
5. **Question-Answering (QA) Generation:** Question-answer pairs were constructed from both aligned and monolingual chunks, covering three task types: (i) Monolingual QA for factual retrieval within a single jurisdiction; (ii) Comparative QA for explicit comparison of regulatory requirements across jurisdictions; and (iii) Parallel QA, in which similar questions were posed independently for each jurisdiction to assess consistency. DeepSeek-V3.2 assisted in drafting question text and answer options. Candidate items and reference answers were constructed from the cited source provisions and were subsequently assessed through the validation framework described in Section 2.3.

#### 2.2.2. Regulatory object and functional taxonomies.

To support subgroup analyses, each candidate item was assigned to one of five primary regulatory object domains according to the principal regulated object and thematic focus of its source documents. Low-frequency or heterogeneous labels that could not be consolidated into the five primary domains were assigned to an additional Other category. This single-label taxonomy was intentionally coarse-grained and was designed for benchmark analysis rather than comprehensive legal classification.

The regulatory object domains were defined as follows:

**Drugs:** Regulation across the full pharmaceutical product lifecycle, including drug registration and approval pathways, clinical trials, good manufacturing practice (GMP), pharmacovigilance and adverse event reporting, labeling and advertising compliance, post-approval changes, and post-market surveillance.
**Medical Devices:** Regulation of medical devices and in vitro diagnostics, covering device classification, registration or filing requirements, quality management systems, including Quality Management System (QMS) requirements, the Quality System Regulation (QSR), and International Organization for Standardization (ISO) 13485, labeling and Unique Device Identification (UDI), post-market adverse event reporting, recalls and corrective actions, clinical evaluation, and manufacturing or import compliance.
**Cosmetics:** Regulation of cosmetic products and ingredients, including product classification, ingredient compliance and restricted substances, efficacy claims and labeling, safety testing requirements, manufacturing and distribution practices, post-market surveillance, and, where applicable, import or cross-border e-commerce regulation.
**General FDA:** Cross-cutting administrative and institutional requirements not limited to a single product category, including definitions, inspection and enforcement, recordkeeping, general procedures, penalties, regulatory communication, and appeals. Functionally related Chinese administrative requirements were included in this domain for comparative analysis.
**Controlled Substances:** Regulation of controlled substances, narcotics, psychotropic drugs, and related chemicals, including licensing and quota systems, storage and security requirements, prescription and distribution controls, transportation and inventory management, reporting obligations, and penalties for non-compliance, as well as requirements relevant to manufacturing or research use.
**Other:** Low-frequency or heterogeneous regulatory categories that could not be reliably consolidated into one of the five primary object domains. This category was retained for descriptive completeness and was not treated as a substantively homogeneous domain.

Separately, each item was assigned at the source-document level to one of seven regulatory functional subdomains: Registration and Approval; Clinical Trials and Good Clinical Practice; Manufacturing and Quality; Non-clinical Studies; Labeling and Packaging; Post-market Surveillance; or General Administration. The functional taxonomy characterized the regulatory activity addressed by the source document and was distinct from the object-domain taxonomy. The distributions of the regulatory object domains and functional subdomains in the final released dataset are reported in the Results section.

### 2.3 Dataset quality validation and release construction

Starting from 11,871 candidate items, we implemented a three-stage quality-validation framework comprising deterministic structural validation, full-dataset semantic quality screening, and risk-stratified expert review. This framework was designed to identify structural incompatibilities, material content errors, and unresolved items before dataset release, while preserving item-level validation and disposition records. It constituted structured dataset quality validation rather than exhaustive provision-by-provision legal verification.

#### 2.3.1. Deterministic structural validation.

All 11,871 candidate items were subjected to deterministic validation rules covering required fields and schema conformity, question and option completeness, reference-answer mapping, identifier and task-language consistency, source-metadata integrity, and compatibility with the strict four-option multiple-choice format. The validation framework comprised rule modules D0-D7, with individual issue codes retained in the item-level audit records. Definitions of the rule modules and issue codes, together with the identifiers of the 12 format-invalid items, are provided in S1 Appendix.

Items failing the strict four-option schema requirements were excluded before semantic screening. This stage identified 12 format-invalid items, leaving 11,859 structurally valid items for subsequent quality assessment.

### 2.3.2. Full-dataset semantic quality screening.

The 11,859 structurally valid items underwent full-dataset semantic screening using five quality dimensions:

1. C1, marked-answer correctness;
2. C2, explanation correctness and consistency;
3. C3, answerability and unique best-answer validity;
4. C4, question formulation and task validity;
5. C5, validity and quality of the cross-jurisdictional comparison.

C1-C4 were applied to all task types, whereas C5 was applied only to Comparative items. For Comparative items, C5 assessed whether the paired regulatory provisions supported the comparison posed and whether their relationship was represented accurately without assuming legal equivalence. Each applicable dimension was assigned one of five values: 2, indicating that the criterion was satisfied; 1, indicating a borderline but non-material concern; 0, indicating a material defect; unable to assess (U); or not applicable (NA).

Semantic screening was performed through the official DeepSeek application programming interface (API) using deepseek-v4-flash. The model was configured with a temperature of 0.0, a maximum output length of 3,200 tokens, a request timeout of 120 seconds, and a maximum of five retry attempts. The complete screening prompt, C1–C5 scoring rubric, output schema, disposition hierarchy, and retry procedure are provided in S1 Appendix.

A predefined disposition hierarchy was applied to the dimension-level ratings. Items with at least one applicable dimension scored as 0 were assigned to the screen-positive pool. Items without a material-error signal and containing only pass or borderline ratings were assigned to the screen-negative population. Items whose substantive status could not be resolved were assigned to the unable/unresolved pool, whereas persistent API or output-schema failures after retry processing were assigned to the technical-unresolved pool.

This procedure produced four post-screening pools: 806 screen-positive items, a screen-negative population of 10,919 items, 126 unable/unresolved items, and 8 technical-unresolved items. The technical-unresolved items were excluded from expert review and from the final release. Embedding similarity was not used to determine regulatory equivalence or item validity; cross-jurisdictional comparison quality was assessed directly through C5.

#### 2.3.3. Risk-stratified expert validation.

Risk-stratified expert validation was conducted by two senior experts with more than 10 years of professional experience in market regulation and compliance within the Shanghai municipal market-regulation system. The experts independently assessed each assigned item using the same C1-C5 framework applied during semantic screening.

The expert review package contained the question, answer options, marked reference answer, explanation, source identifiers, and provision-level metadata. These materials enabled the experts to locate and consult the original regulatory text when necessary, although complete source documents were not embedded in every review package. Accordingly, the procedure was designed as structured expert validation of item quality and regulatory consistency rather than exhaustive independent legal verification of every provision represented in the dataset.

Three complementary expert-review cohorts were constructed:

- Cohort A: all 806 screen-positive items identified during semantic screening;
- Cohort B: a stratified random sample of 500 items drawn from the screen-negative population of 10,919 items; and
- Cohort C: all 126 unable/unresolved items.

Cohort B was stratified by task type and language, producing six task-by-language strata. Sample sizes were allocated using the Hamilton method, and sampling was performed using a fixed random seed of 2025. In total, 1,432 items underwent independent dual-expert validation.

Inter-rater agreement was assessed at both the dimension level and the final retain-versus-exclude disposition level, as described in the Statistical Analysis section. Reviewer-specific ratings and item-level validation records were retained to support reproducibility and auditability.

#### 2.3.4. Final quality disposition and release construction.

Following independent expert review, the reviewer-specific records were reconciled programmatically according to a predefined severity-based procedure. An item was classified as a material error and excluded if either expert identified a major correction on any applicable quality dimension. Items without a major-correction rating were retained unless they remained unresolved after reconciliation. The reviewer-level aggregation rules and complete reconciliation matrix are provided in S1 Appendix. Final candidate-item dispositions were classified as pass, borderline, material error, unable to assess, technical unresolved, or format invalid.

Items assigned a final pass designation contained no identified quality defect. Borderline items contained one or more minor or non-material concerns but retained an answerable question, a unique and defensible best answer, and an explanation consistent with that answer. Both pass and borderline items were retained in the final dataset. Items classified as material error, unable to assess, technical unresolved, or format invalid were excluded. Screen-negative items not selected for expert review were retained according to their prespecified semantic-screening disposition.

The resulting expert-review outcomes, exclusion counts, residual-risk estimate, and final release composition are reported in Section 3.1.

### 2.4. Models evaluated

We selected four large language models from different providers to establish a multi-provider evaluation baseline. Model selection considered their relevance to English–Chinese bilingual regulatory tasks and their availability through official application programming interfaces (APIs), enabling evaluation under a standardized protocol. All models were accessed through their respective official APIs on 22 July 2026, using the following exact model identifiers:

The evaluated models were:

1. GPT (gpt-5.6-terra; OpenAI);
2. Gemini (gemini-3.6-flash; Google);
3. DeepSeek (deepseek-v4-flash; DeepSeek);
4. Qwen (qwen-3.5-max; Alibaba Cloud).

Together, these models provide a multi-provider baseline for comparing performance under a common evaluation protocol, rather than exhaustive coverage of available LLMs.

### 2.5. Evaluation protocol

To support reproducible evaluation, we developed a standardized evaluation harness informed by practices used in large-scale LLM benchmarks, including Massive Multitask Language Understanding (MMLU) [16]. The harness applied a common prompt structure, predefined JavaScript Object Notation (JSON)-based parsing rules, and a uniform zero-shot evaluation protocol across all models. The evaluation code, final dataset release, prompt templates, and scoring scripts are hosted on GitHub.

Before the main analysis, 10 randomly selected items were used in a pilot run solely to test prompt formatting and compatibility with the predefined JSON parser. Pilot outputs were not used for performance estimation. After the prompt and output schema were finalized, all four models were evaluated on the complete final release of 11,444 items under the same protocol.

#### 2.5.1. Prompting and output parsing.

We employed a standardized, instruction-based prompting strategy designed to minimize output variability and ensure reproducible evaluation across models. The complete prompt templates, including system instructions, user input structure, and strict JSON output schema, are provided in S1 Appendix to support replication of the evaluation protocol. All models were evaluated under a zero-shot setting, in which only the target question and its multiple-choice options were provided.

Models were instructed to assume the role of a regulatory expert familiar with US FDA and Chinese NMPA regulations, analyze the regulatory context, and select the most appropriate answer from the given options. To facilitate automated parsing and downstream evaluation, responses were constrained to a strict JSON schema specifying the selected option and a brief textual justification.

For all evaluations, the temperature was set to 0.0, the top-p parameter was set to 1.0, the maximum output length was set to 1,024 tokens, the request timeout was 90 seconds, and up to five retry attempts were permitted. These settings were applied consistently across the four official model application programming interfaces.

Only the parsed selected-answer field was used to determine correctness. The generated justification was not treated as a study outcome and was retained only for audit and debugging purposes. An output was considered valid only when it could be parsed into the required JSON structure and contained a recognizable answer option. Outputs that remained invalid after retry processing, including malformed JSON, missing selected-answer fields, or answers that could not be mapped to the available options, were counted as incorrect.

### 2.6. Statistical analysis

Statistical analyses were implemented using Python scripts. Descriptive statistics were presented as counts and percentages for categorical variables. The primary outcome measure was accuracy, defined as the number of correct responses divided by the fixed evaluation denominator of 11,444 items. API failures, parsing failures, and outputs without a valid predicted answer remained in the denominator and were scored as incorrect.

Pairwise differences in model accuracy were assessed using two-sided exact mid-P McNemar tests on paired predictions across the complete final release (n = 11,444). Six pairwise comparisons were conducted, and p values were adjusted using the Holm–Bonferroni step-down procedure. Matched-pairs odds ratios (ORs) and their 95% confidence intervals (CIs) were calculated from discordant prediction pairs. Model pairs were ordered with the higher-accuracy model listed first, such that an OR greater than 1 favored the first-listed model.

Agreement between the two expert evaluators was assessed using exact agreement and chance-corrected measures. For C1–C4, applicable paired ratings were dichotomized as material issue (score 0) versus no material issue (scores 1 or 2), and binary Cohen's κ with 95% CIs was calculated [17]. Ratings coded as unable to assess or not applicable were excluded from the corresponding dimension-specific calculation. For C5, which applied only to Comparative items and showed marked prevalence imbalance, exact agreement and prevalence-adjusted bias-adjusted kappa (PABAK) were used as the primary measures; binary Cohen's κ was reported as a supplementary measure. Agreement on the final retain-versus-exclude disposition was assessed using binary Cohen's κ after excluding items without paired binary dispositions.

For Cohort B, the residual material-error proportion in the semantic screen-negative population was summarized using the observed sample proportion and a Wilson 95% CI. All statistical tests were two-sided, and a Holm-adjusted p value below 0.05 was considered statistically significant.

## 3. Results

### 3.1. Dataset validation and release outcomes

**Fig 2:**
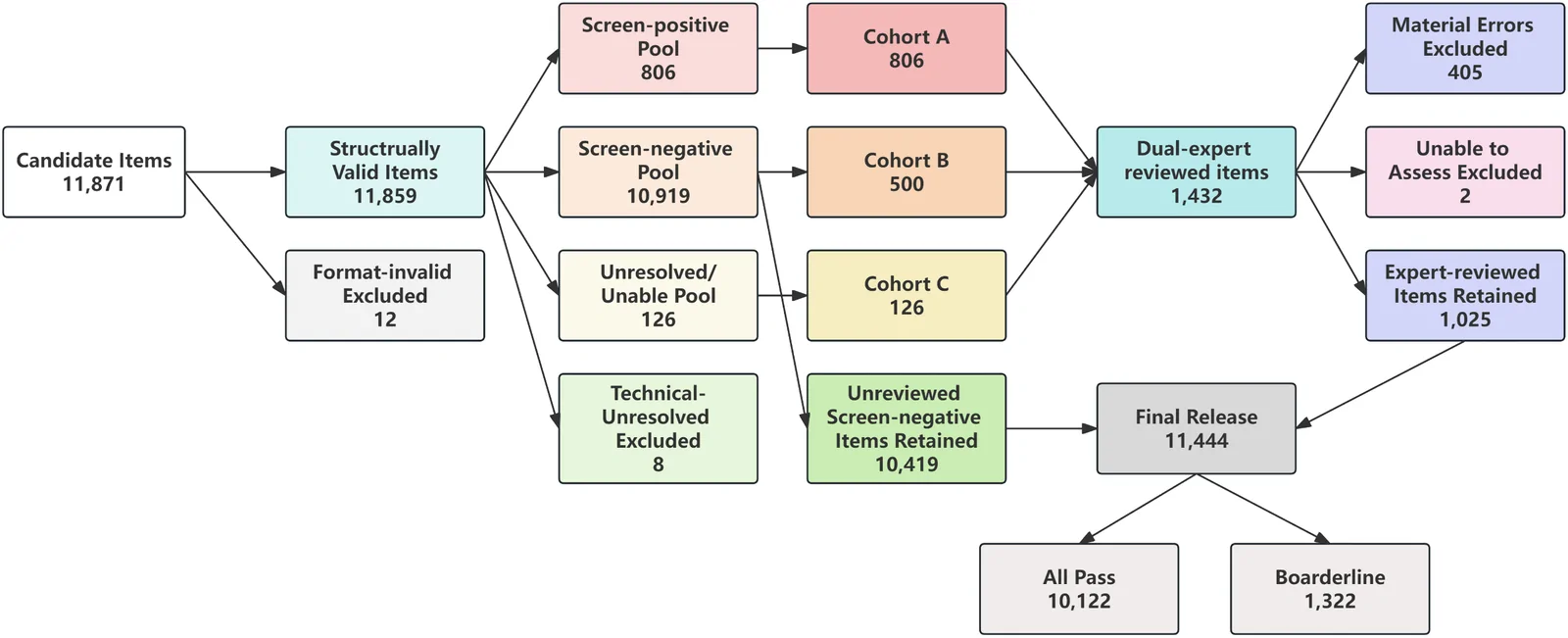
Validation and release construction workflow for Sino-US-DrugQA.

The validation and release process is summarized in Fig 2. Of the 11,871 candidate question–answer items, 12 failed deterministic structural validation because they were incompatible with the strict four-option multiple-choice format. The remaining 11,859 structurally valid items underwent full-dataset semantic quality screening across the C1–C5 dimensions.

Semantic screening assigned 806 items to the screen-positive pool, 10,919 to the screen-negative pool, 126 to the unable/unresolved pool, and 8 to the technical-unresolved pool. All 806 screen-positive items (Cohort A), a stratified random sample of 500 screen-negative items (Cohort B), and all 126 unable/unresolved items (Cohort C) underwent independent review by two market-regulation and compliance experts, resulting in a total expert-reviewed sample of 1,432 items.

Of the 1,432 expert-reviewed items, 1,025 were retained, 405 were excluded because of material errors, and 2 were excluded as unable to assess. The remaining 10,419 screen-negative items that were not sampled for expert review were retained following semantic screening, whereas the 8 technical-unresolved items were excluded. The final Sino-US-DrugQA release therefore comprised 11,444 items. Of these, 10,122 were classified as pass and 1,322 as borderline. In total, 427 candidate items were excluded: 12 format-invalid items, 405 items with material errors, 2 items that could not be assessed, and 8 technical-unresolved items.

Within Cohort B, expert review identified material errors in 18 of the 500 sampled screen-negative items, corresponding to an observed residual material-error proportion of 3.60% (Wilson 95% confidence interval [CI], 2.29%–5.62%). This estimate characterizes residual risk within the sampled screen-negative population and should not be interpreted as the error rate of the complete final release.

Inter-expert agreement varied across the five quality dimensions (Table 3). For C1–C4, exact agreement ranged from 88.86% to 95.31%, and binary Cohen's κ ranged from 0.446 to 0.578. For cross-jurisdictional comparison quality (C5), exact agreement was 72.50%, and the prevalence-adjusted bias-adjusted kappa (PABAK) was 0.450. Agreement on the final retain-versus-exclude disposition was 75.79%, with a binary Cohen's κ of 0.453 among items with paired binary dispositions.

**Table 3:** Inter-expert agreement during risk-stratified quality validation.

Assessment criterion	Paired n	Exact agreement, % (95% CI)	Chance-corrected agreement
C1: Marked-answer correctness	1,217	92.77 (91.18–94.09)	Cohen's κ = 0.578 (0.493–0.663)
C2: Explanation correctness and consistency	1,242	90.82 (89.09–92.30)	Cohen's κ = 0.530 (0.493–0.567)
C3: Answerability and unique best-answer validity	1,257	95.31 (93.99–96.34)	Cohen's κ = 0.519 (0.473–0.565)
C4: Question formulation and task validity	1,319	88.86 (87.04–90.44)	Cohen's κ = 0.446 (0.337–0.554)
C5: Cross-jurisdictional comparison quality	651	72.50 (68.95–75.79)	PABAK = 0.450
Final retain-versus-exclude disposition	1,268	75.79 (73.36–78.07)	Cohen's κ = 0.453 (0.408–0.497)

C5 was applicable only to Comparative items. Because the C5 ratings exhibited marked prevalence imbalance, exact agreement and PABAK were treated as the primary agreement measures; binary Cohen's κ was reported as a supplementary measure and was 0.312 (95% CI, 0.204–0.420). For each dimension, analyses included only items with paired applicable ratings; ratings coded as unable to assess or not applicable were excluded. Items without paired binary retain-versus-exclude dispositions were excluded from the final-disposition agreement analysis.

### 3.2. Characteristics of the final released dataset

The final Sino-US-DrugQA release comprised 11,444 multiple-choice question–answer items. Table 4 summarizes their distribution by task type, language, and regulatory object domain.

**Table 4:** Characteristics of the final Sino-US-DrugQA release.

Characteristic	n	Percentage (%)
Total QA Pairs	11,444	100
Monolingual	6,903	60.32
Comparative	4,003	34.98
Parallel	538	4.70
Language		
English	5,753	50.27
Chinese	5,691	49.73
Regulatory Domain		
Drugs	4,571	39.94
Medical Devices	2,683	23.44
Cosmetics	1,650	14.42
General FDA	1,401	12.24
Controlled Substances	819	7.16
Other	320	2.80

Monolingual QA accounted for 6,903 items (60.32%), while Comparative and Parallel QA comprised 4,003 (34.98%) and 538 (4.70%) items, respectively. The release was approximately balanced by language, with 5,753 English-language items (50.27%) and 5,691 Chinese-language items (49.73%).

Drugs was the largest regulatory object domain, comprising 4,571 items (39.94%), followed by Medical Devices (23.44%) and Cosmetics (14.42%). The Other category contained 320 items (2.80%) representing heterogeneous low-frequency labels that could not be consolidated into the five primary domains.

### 3.3. Overall performance

All four models were evaluated under the same zero-shot protocol on the final 11,444-item release. Accuracy was calculated using the fixed denominator of 11,444 items; API failures, parsing failures, and outputs without a valid predicted answer were scored as incorrect. Valid-output coverage was reported separately to describe output-format reliability. Overall zero-shot performance is summarized in Table 5.

**Table 5:** Overall zero-shot performance of the four evaluated models on the final Sino-US-DrugQA release.

Model	Correct responses, n	Accuracy, %	Valid-output coverage, %	Parsing failures, n
GPT (gpt-5.6-terra)	9,891	86.43	100.00	0
Gemini (gemini-3.6-flash)	9,828	85.88	99.07	106
DeepSeek (deepseek-v4-flash)	9,757	85.26	99.88	14
Qwen (qwen-3.5-max)	9,523	83.21	99.95	6

GPT achieved the highest observed overall accuracy at 86.43%, followed by Gemini at 85.88%, DeepSeek at 85.26%, and Qwen at 83.21%. The observed differences from GPT were 0.55 percentage points for Gemini, 1.17 percentage points for DeepSeek, and 3.22 percentage points for Qwen. Pairwise significance tests are reported in Section 3.6.

Valid-output coverage exceeded 99% for all four models. GPT produced a valid, parseable response for every item, whereas Gemini, DeepSeek, and Qwen produced 106, 14, and 6 parsing failures, respectively. Because invalid outputs remained in the fixed denominator and were scored as incorrect, no evaluated items were removed from the accuracy calculations.

### 3.4. Performance by task type and language

**Fig 3:**
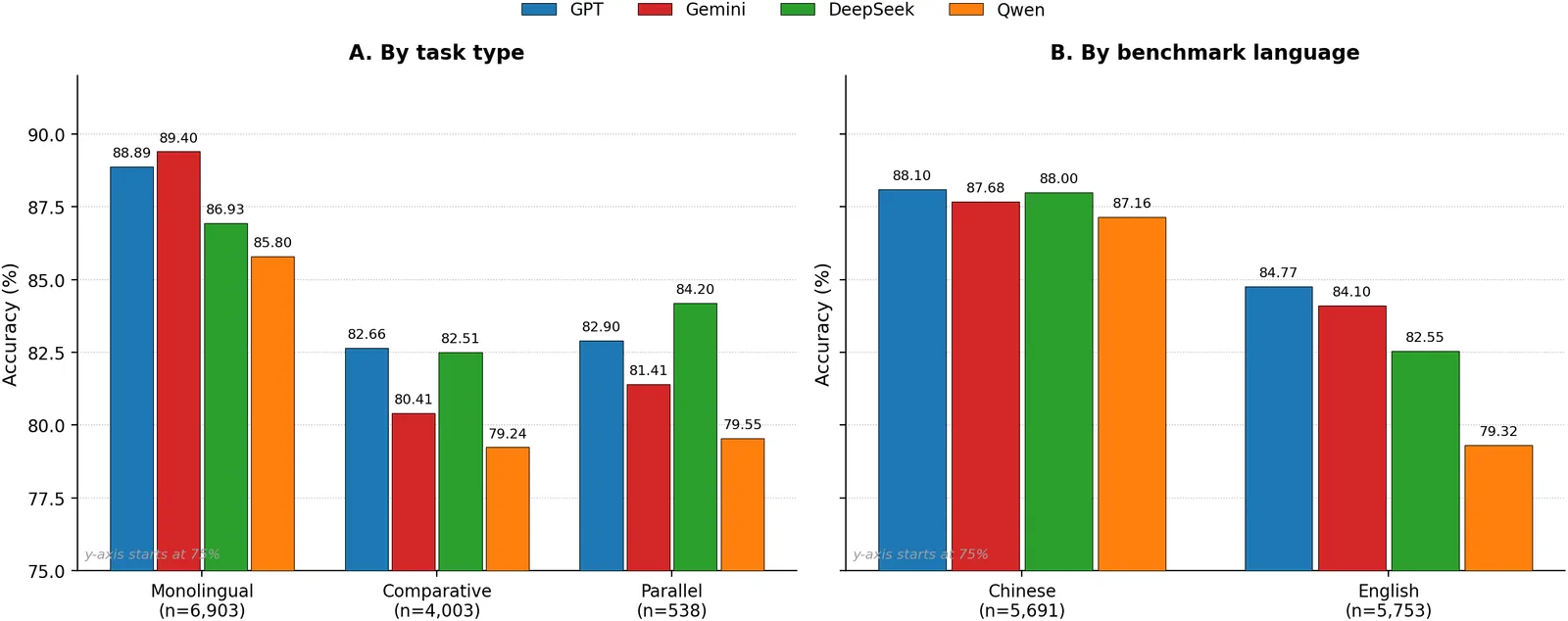
Zero-shot model accuracy by task type and benchmark language.

Model performance by task type and benchmark language is summarized in Fig 3 and Table 6. Across all four models, accuracy was lower on Comparative QA than on Monolingual QA.

**Table 6:** Zero-shot accuracy by task type and language.

Model	Monolingual, %	Comparative, %	Parallel, %	Chinese, %	English, %
GPT (gpt-5.6-terra)	88.89	82.66	82.90	88.10	84.77
Gemini (gemini-3.6-flash)	89.40	80.41	81.41	87.68	84.10
DeepSeek (deepseek-v4-flash)	86.93	82.51	84.20	88.00	82.55
Qwen (qwen-3.5-max)	85.80	79.24	79.55	87.16	79.32

The Comparative–Monolingual accuracy difference was 4.42 percentage points for DeepSeek, 6.23 for GPT, 6.56 for Qwen, and 8.98 for Gemini. Although its magnitude varied, the direction of the difference was consistent across models. Accuracy on the smaller Parallel subset ranged from 79.55% to 84.20% and is reported descriptively.

All four models also achieved higher accuracy on Chinese-language than on English-language items. The observed language differences ranged from 3.33 percentage points for GPT to 7.84 percentage points for Qwen. These subgroup comparisons were descriptive and should not be interpreted as independent causal effects of task type, language, or jurisdiction.

### 3.5. Performance by regulatory object domain and functional subdomain

**Fig 4:**
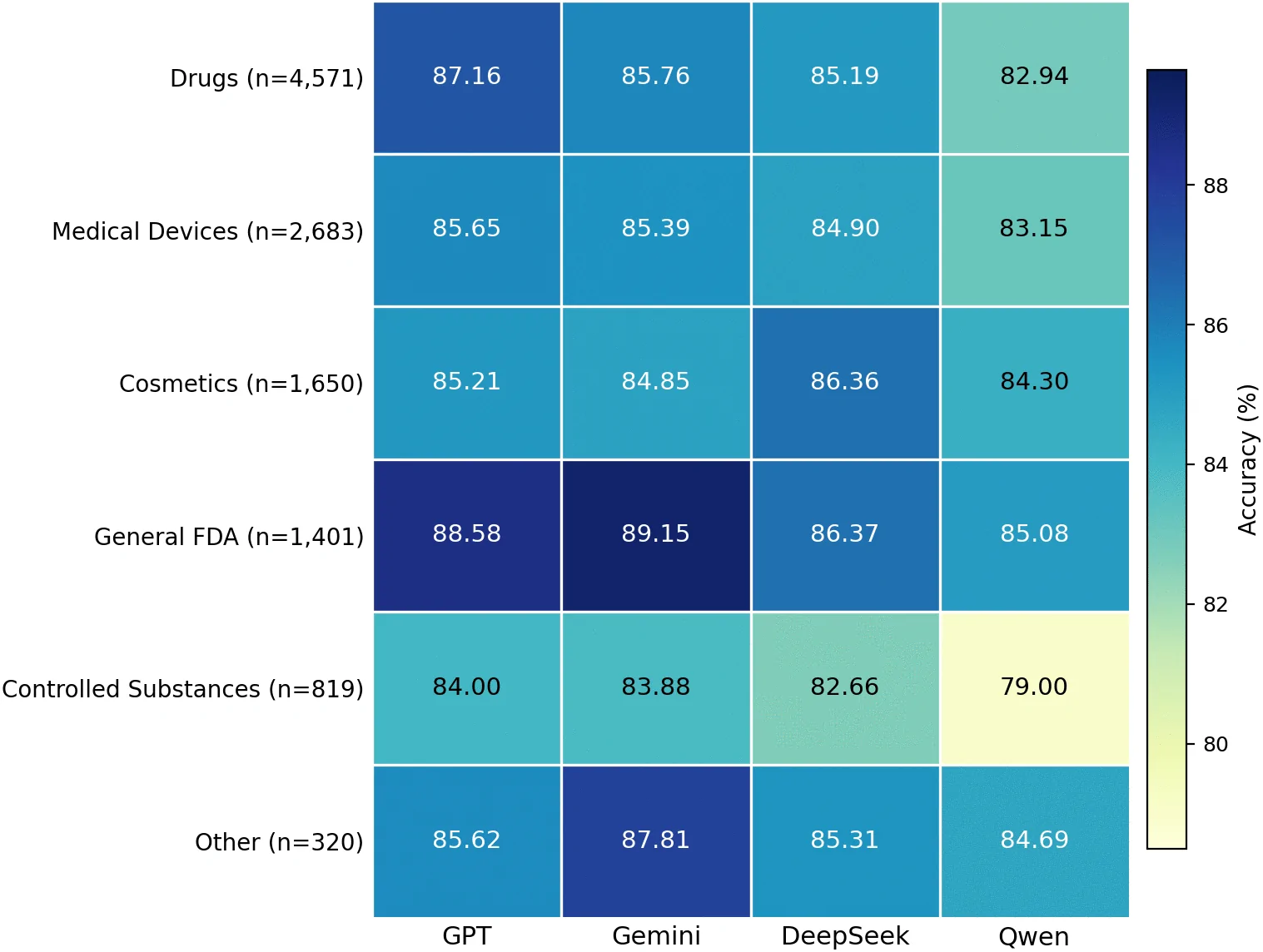
Heatmap of zero-shot model accuracy across regulatory object domains in the final Sino-US-DrugQA release.

Model performance was examined across the five primary regulatory object domains and the additional Other category defined in Section 2.2. The object-domain results are shown in Fig 4.

Controlled Substances had the lowest observed accuracy among the five primary domains for all four models, ranging from 79.00% for Qwen to 84.00% for GPT. General FDA had the highest observed domain-specific accuracy for for all four models. Performance on Medical Devices varied within a comparatively narrow range of 83.15%–85.65%. The Other category comprised heterogeneous low-frequency labels and was included for descriptive completeness rather than interpreted as a homogeneous regulatory domain.

Performance was additionally assessed across the seven predefined regulatory functional subdomains. Table 7 reports the subgroup size and zero-shot accuracy for each model.

**Table 7:** Zero-shot accuracy of four evaluated large language models across seven regulatory functional subdomains.

Functional subdomain	n	GPT, %	Gemini, %	DeepSeek, %	Qwen, %
Registration and Approval	3,960	84.72	84.07	83.51	81.01
Clinical Trials and GCP	526	90.11	88.21	86.50	86.69
Manufacturing and Quality (GMP)	2,573	86.28	86.24	86.67	83.75
Non-clinical Studies (GLP)†	22	81.82	95.45	90.91	81.82
Labeling and Packaging	952	86.45	84.66	82.88	79.52
Post-market Surveillance	607	84.51	84.35	84.35	83.53
General Administration	2,804	88.73	88.34	87.16	86.38

†The Non-clinical Studies subgroup contained only 22 items; its estimates are reported for descriptive purposes and should not be used to draw comparative conclusions.

Among the adequately represented functional subdomains, the highest observed accuracies generally occurred in Clinical Trials and GCP and General Administration. GPT achieved the highest observed accuracy in five subdomains, whereas DeepSeek achieved the highest accuracy in Manufacturing and Quality. Model accuracies were most similar in Post-market Surveillance, ranging from 83.53% to 84.51%, and showed a wider spread in Labeling and Packaging, ranging from 79.52% to 86.45%.

Because these subgroup analyses covered all items assigned to each category in the final release, the reported percentages are descriptive benchmark results rather than sample-based population estimates.

### 3.6. Pairwise comparisons of overall model accuracy

Pairwise differences in model accuracy were assessed using two-sided exact mid-P McNemar tests on paired predictions across the fixed evaluation set of 11,444 items. Six pairwise comparisons were conducted, and p values were adjusted using the Holm–Bonferroni procedure. In each comparison, the model with the higher observed overall accuracy was listed first; thus, an odds ratio (OR) greater than 1 favored the first-listed model. The results of these pairwise comparisons are summarized in Table 8.

**Table 8:** Pairwise comparisons of model performance using exact mid-P McNemar tests.

Model comparison	OR	95% CI	Holm-adjusted p value
GPT vs Gemini	1.102	0.988–1.229	0.1488
GPT vs DeepSeek	1.175	1.067–1.293	0.0031
GPT vs Qwen	1.540	1.398–1.695	<0.001
Gemini vs DeepSeek	1.094	0.991–1.207	0.1488
Gemini vs Qwen	1.449	1.314–1.597	<0.001
DeepSeek vs Qwen	1.366	1.234–1.513	<0.001

ORs were calculated from discordant paired outcomes. Because the higher-accuracy model was listed first, an OR greater than 1 indicates that the first-listed model was more frequently correct when the paired predictions differed.

No statistically significant difference was detected between GPT and Gemini after Holm adjustment (OR = 1.102, 95% CI 0.988–1.229; adjusted p = 0.1488), or between Gemini and DeepSeek (OR = 1.094, 95% CI 0.991–1.207; adjusted p = 0.1488).

GPT had significantly better paired outcomes than DeepSeek (OR = 1.175, 95% CI 1.067–1.293; adjusted p = 0.0031) and Qwen (OR = 1.540, 95% CI 1.398–1.695; adjusted p < 0.001). Gemini and DeepSeek also had significantly better paired outcomes than Qwen (OR = 1.449 and 1.366, respectively; both adjusted p < 0.001). Overall, four of the six pairwise comparisons remained statistically significant after correction for multiple testing.

## 4. Discussion

### 4.1. Principal findings and quality of the final dataset release

The principal contribution of this study is the development of a bilingual benchmark for cross-jurisdictional pharmaceutical regulation supported by a structured, risk-stratified validation process. From 11,871 candidate items, structural validation, full-dataset semantic screening, and dual-expert review produced a final release of 11,444 items, with 427 structurally invalid, materially flawed, or unresolved items excluded. This process demonstrates both the scalability of LLM-assisted dataset construction and the continuing need for expert quality control.

Because the expert-reviewed set deliberately overrepresented screen-positive and unresolved items, the 405 material errors identified among the 1,432 reviewed items should not be interpreted as a dataset-wide error rate. Cohort B provided a more relevant indication of residual risk within the screen-negative population: 18 of 500 sampled items were classified as material errors, corresponding to 3.60% (Wilson 95% CI, 2.29%–5.62%). Semantic screening therefore reduced, but did not eliminate, the risk of material defects among retained items.

Inter-expert agreement was higher for the relatively concrete C1–C4 criteria, with exact agreement ranging from 88.86% to 95.31%, than for cross-jurisdictional comparison quality (C5), for which exact agreement was 72.50%. The lower C5 agreement reflects the greater interpretive difficulty of evaluating comparisons between non-equivalent regulatory systems. The moderate chance-corrected agreement measures also support the continued use of explicit decision rules, reviewer calibration, and transparent reconciliation procedures.

The final release contained 10,122 pass items and 1,322 borderline items. Borderline items were retained because their identified concerns were non-material and the questions remained answerable with a unique and defensible best answer. Preserving the pass–borderline distinction allows users to retain broader benchmark coverage or apply a more conservative quality threshold according to their analytical purpose.

The validation should nevertheless be understood as structured expert assessment rather than exhaustive legal verification of every item. Experts reviewed the questions, answer options, reference answers, explanations, and source metadata, which allowed the original regulatory provisions to be consulted when necessary, although complete source documents were not embedded in every review package. In addition, embedding similarity was used only to retrieve candidate provision pairs and did not establish legal equivalence; comparative-item validity was assessed separately through C5. Sino-US-DrugQA should therefore be regarded as a quality-controlled but imperfect benchmark, and individual answers should be verified against current primary sources before use in high-stakes regulatory contexts.

### 4.2. Benchmark positioning and contribution to regulatory science

Relative to existing legal and medical question-answering benchmarks, Sino-US-DrugQA addresses a distinct evaluation setting. LawBench [11] and LexGLUE [12] primarily assess legal understanding and reasoning within individual legal systems, whereas MedMCQA [13] focuses on clinical and biomedical knowledge. Sino-US-DrugQA instead evaluates bilingual, provision-level questions involving administrative requirements across the non-equivalent pharmaceutical regulatory systems of the US Food and Drug Administration and China's National Medical Products Administration.

The final release combines 6,903 Monolingual, 4,003 Comparative, and 538 Parallel items, enabling separate assessment of single-jurisdiction questions, explicit cross-jurisdictional comparisons, and related questions posed independently within the two regulatory systems. Although these task categories do not isolate the causes of individual model errors, they provide a standardized basis for comparing performance across different regulatory-reasoning demands. The benchmark also covers regulatory activities across the pharmaceutical product lifecycle. Its object-domain and functional-subdomain taxonomies support analysis by both regulated product and regulatory function, two complementary dimensions that may cut across jurisdictional frameworks.

Item-level source metadata and publicly accessible regulatory references support provision-level review and correction of benchmark items. The public repository provides the stable dataset version, evaluation harness, and analysis resources corresponding to this study, thereby supporting reproducible use of the reported benchmark. Because the dataset reflects regulatory documents valid as of December 2025, its results should be interpreted within that temporal scope. Users should verify the applicable version of the primary regulatory sources before using individual items beyond benchmarking or research.

### 4.3. Interpretation of model performance across tasks, languages, and regulatory domains

Across all four models, Comparative QA accuracy was 4.42–8.98 percentage points lower than Monolingual QA accuracy. This consistent gap is compatible with the additional demands of identifying related provisions across two non-equivalent regulatory systems and determining how their requirements differ. Parallel QA also yielded lower accuracy than Monolingual QA, suggesting that performance may vary when related regulatory issues are framed through different jurisdiction-specific terminology and administrative structures. However, the present design did not separately measure retrieval, translation, regulatory concept alignment, or comparative deduction, and therefore cannot identify the specific mechanism underlying these differences.

All four models achieved higher accuracy on Chinese-language than on English-language items, with differences of 3.33–7.84 percentage points. These results should not be interpreted as evidence of intrinsically stronger Chinese-language competence because the two language subsets also differed in jurisdiction, source materials, task composition, domain distribution, and potentially item difficulty. Although translation ability is relevant to multilingual question answering [18], linguistic variation alone is unlikely to account for the Comparative–Monolingual gap, which also involves differences in legal scope, responsible authorities, procedures, and compliance thresholds.

Performance also varied across regulatory domains. Controlled Substances had the lowest observed accuracy for all four models, whereas General FDA had the highest accuracy for all four models. Across functional subdomains, accuracies were generally high in Clinical Trials and Good Clinical Practice and General Administration, while Labeling and Packaging showed greater variation across models. Shared terminology arising from international harmonization frameworks may contribute to performance in some functional areas [19,20], but the observational subgroup analysis does not establish a causal relationship. The Non-clinical Studies subgroup contained only 22 items and does not support stable cross-model conclusions.

Overall, benchmark performance reflected the combined influence of task structure, language, regulatory object, and regulatory function. The results identify consistent areas of relative difficulty, particularly cross-jurisdictional comparison, but controlled analyses would be required to distinguish the contributions of translation, source retrieval, concept alignment, and comparison-specific reasoning.

### 4.4. Implications for expert-supervised regulatory use

Although the four evaluated models achieved overall accuracies of 83.21%–86.43%, none was fully reliable, and all performed less accurately on Comparative than on Monolingual items. These findings do not directly quantify real-world compliance risk, but they indicate that model outputs may contain jurisdictional, procedural, or provision-level errors that require independent verification. This need for governance and professional oversight is consistent with broader recommendations for generative artificial intelligence in safety-critical healthcare applications [8,9].

LLMs should therefore be positioned as tools within expert-supervised regulatory workflows rather than as autonomous sources of regulatory interpretation. They may assist with preliminary retrieval, identification of relevant provisions, and drafting of cross-jurisdictional comparisons, while regulatory professionals remain responsible for verifying source authenticity and temporal validity, determining legal applicability, resolving non-equivalent concepts, and making final compliance decisions. Potential safeguards include retrieval from authoritative version-controlled sources, automated checking of citations and provision identifiers, explicit handling of uncertain or invalid outputs, and mandatory expert review of comparative conclusions. The effectiveness of these safeguards was not evaluated in this study and requires deployment-oriented assessment.

The reported results should also not be interpreted as evidence that model performance is equivalent to human regulatory expertise. No human baseline was established under the same benchmark conditions, and benchmark accuracy does not capture the full administrative, institutional, temporal, and legal context of professional regulatory judgment. Sino-US-DrugQA is therefore intended to evaluate the capabilities and limitations of model-assisted regulatory retrieval and comparison under expert supervision, rather than to determine which regulatory decisions can be automated.

### 4.5. Limitations and future directions

This study has several limitations. First, candidate generation, concept extraction, provision alignment, and semantic screening partly relied on automated language-model pipelines. Although independent expert review was incorporated, correlated model biases may remain. Expert validation was also risk-stratified rather than exhaustive; therefore, residual errors may persist among screen-negative items retained without expert review. Automated screening should be understood as a scalable risk-detection mechanism rather than a substitute for independent regulatory verification.

Second, the validation process assessed item quality but did not constitute exhaustive legal verification of every reference answer. Experts reviewed the item content and source metadata and could consult the cited provisions when necessary, but regulatory interpretation may also depend on definitions, exceptions, implementation guidance, and factual context beyond the linked provision. The lower agreement observed for cross-jurisdictional comparison quality further indicates that comparisons between non-equivalent regulatory systems remain partly interpretive. Future validation could use more extensive reviewer calibration, provision-level source materials, and adjudication of disputed cases.

Third, the final release retained borderline items with non-material wording, presentation, or explanation concerns. Their inclusion preserved regulatory and task coverage, but users requiring a more conservative quality threshold may restrict analyses to items labeled as pass. In addition, the regulatory taxonomies simplify questions that may span multiple functions, and the Other object-domain category remains heterogeneous. Question-level multi-label annotation could improve future versions.

Fourth, the evaluation included four API-accessed models under one zero-shot protocol and at a single evaluation date. The findings may not generalize to other model versions, prompting strategies, retrieval-grounded systems, tool-assisted workflows, or locally deployed models. Because no human-performance baseline was established under comparable conditions, the reported accuracies should not be interpreted as equivalence to expert regulatory judgment.

Finally, the benchmark reflects regulatory materials valid as of December 2025. Subsequent amendments, guidance, or changes in regulatory practice may affect individual items. The present release should therefore be interpreted as a versioned benchmark with a defined temporal scope, and users should verify current primary sources when applying its content beyond research evaluation.

## 5. Conclusions

This study introduces Sino-US-DrugQA, a bilingual benchmark for evaluating large language models in cross-jurisdictional pharmaceutical regulation between the United States and China. Structural validation, full-dataset semantic screening, and risk-stratified expert review produced a final validated release of 11,444 items. Item-level regulatory metadata and publicly available evaluation and analysis resources support transparent and reproducible research on bilingual regulatory question answering.

Across the four evaluated models, overall zero-shot accuracy ranged from 83.21% to 86.43%, and Comparative accuracy was consistently 4.42–8.98 percentage points lower than Monolingual accuracy. These results identify cross-jurisdictional comparison as an area of relative difficulty but do not establish that current models are suitable for autonomous regulatory interpretation or compliance decision-making. Sino-US-DrugQA is intended as a quality-controlled, temporally defined benchmark for evaluating model-assisted regulatory retrieval and comparison. In high-stakes applications, model outputs should remain subject to expert review and verification against authoritative, current regulatory sources.

## Supporting information

S1 Appendix Dataset validation and model evaluation specifications. (DOCX)
